# Uniformity of State Regulations as to State Examining Boards

**Published:** 1897-11

**Authors:** W. Horace Hoskins

**Affiliations:** Philadelphia, Pa.


### UNIFORMITY OF STATE REGULATIONS AS TO STATE EXAMINING BOARDS.[1]

By W. Horace Hoskins, D.V.S.,
PHILADELPHIA, PA.

In presenting to you this brief review of the efforts put forth during the past year to secure, by State enactment, such legislation as would best conserve the interests of the profession in the several commonwealths of our country, and thus ultimately lead to some single plan embracing a national supervision of veterinary-degree conferring power, I have but little to offer you as the outcome of of this work, but will draw your attention to some of the obstacles preventing the attainment of our wishes.

The following States, during the past year, presented to their Legislatures bills looking to registration of all practitioners of veterinary science and the granting of licenses to all future practitioners wishing to enter upon practice in these States:

*Colorado.* A proposed bill for the appointment of a board of examiners, also the registration, examination, and licensing of graduates; non-graduates able to prove having practised in the State for five years previous to the passage of the law, allowed to register within ninety days after the bill has been adopted. Failed to pass. Reasons for the same, lack of united influences of veterinarians; insufficient number of veterinarians in the State; a legislative deadlock at the end of the session.

*New Hampshire.* A proposed act requires registration of veterinarians after July 1, 1897. Satisfactory examination required before an appointed board of examiners, consisting of three persons who must be members of the State association. This bill failed for no other special reason than lack of united effort of the veterinarians of the State.

*Indiana.* Desired the consideration of a statute to regulate the practice of veterinarians. This bill failed.

*Minnesota.* The bill providing for a board of examiners was revised, and must now be composed of legally authorized graduates from recognized schools; graduates must submit their diplomas, and must pass any required examination before the board; non-graduates not permitted to appear for examination. These amendments to the law in force in Minnesota make their provisions

[1] Presented to the Association of Faculties of the Veterinary Colleges of North America at Nashville, September, 1897.

among the best in the land for the encouragement of higher veterinary education.

*Connecticut.* Bill for registration and establishment of a State board of examiners. Defeated by lower branch of the Legislature. Lack of united support among the veterinarians; the State society not strong enough or sufficiently united.

*Michigan.* State Legislature failed to pass the bill to provide for a State board of veterinary medical examiners and better regulation of the practice of veterinary medicine. The same causes operated here—lack of organized support, and those directed to present this legislation had not the advantages of a needed organized profession back of them.

*Wisconsin.* Bill to regulate the practice of veterinary medicine and surgery and dentistry. Only graduates of legally chartered schools can register, others must pass examination before an appointed board of examiners. This bill failed to pass, in part because its provisions, while desirable, were too stringent, and again, lack of hard work on the part of those most interested.

*North Carolina.* A bill to regulate the practice of veterinary medicine and surgery. To prevent delay and inconvenience, two members of the board of examiners may grant a temporary certificate to practise veterinary medicine or surgery, which shall be in force only until the next regular meeting of the board of examiners, but in no case shall such temporary certificate be granted to any person who has been an unsuccessful applicant for a certificate before the board.

In all these States, except Minnesota, the efforts proved fruitless of results, and I shall review briefly the reasons therefor. In each of them, under your direction, I placed myself in communication with those directing these movements, and, so far as possible, secured the adoption of a general plan, consisting of an appointed board of examiners of graduates of recognized schools, and that said board would also act as a board of registration. I endeavored o have eliminated from these proposed statutes any parts that would prevent future recognition of licenses granted by one board among the several others, and in some was able to secure the insertion of provisions that would tend toward a central board of examiners.

Now, in general, we find that the chief causes for lack of success may be summed up under the following heads:

1. Lack of thorough organization of the profession in the several States.

2. Indifference of those who should be most interested in the securing of such safeguards.

3. Lack of public education as to the scope and importance of the science of veterinary medicine.

4. Want of a central directing force, to give impetus and support to these movements.

Now, permit me to say a few words about these several chief causes, that we may better understand the conditions and consider the remedies required.

First, in almost every State there are from four to ten non-graduates, or self-made practitioners, to every graduate. The former are more potential in defeating legislation than the latter are in securing it, because, generally, they have been longer residents of the State, and look with suspicion upon every movement that seems to be directed by college graduates.

Now, as I am firmly convinced that no legislation can be secured in any State that will rob these men of their means of a livelihood, for such legislation is contrary to the "bill of rights" of our land, I think it would be well for the profession in every State, before the final drafting of proposed legislation, to call together a few of the best non-graduates and submit to them the plan of action, and thus disarm, in great measure, the hostility of these men, which is ofttimes the outcome of ignorance and misconception of the purposes and aims of such legislation.

I am opposed to all legislation that has for its aim only the registration of practitioners throughout the State by any county officer, whose source of remuneration is ofttimes by fees only, and thus an incentive to register large numbers presents itself, and little consideration is given to the spirit and purpose of such laws. I am only favorable to legislation that will place registration in the hands of a board of examiners; therefore, in efforts to secure this recognition, there must be a more conservative spirit among all legitimate practitioners of veterinary science, and, before such efforts are put forth, a more thorough organization of the entire profession. We have found in some States many graduates arrayed against proposed legislation, because in some unimportant feature it failed to meet their own views, and these narrow, selfish fellows, raising themselves against the broader interests to be conserved, have sacrificed the chances of successful legislation. In most instances we have found these same members of the profession unwilling and indifferent enough to deny those who were planning these measures their views and assistance in conference as to the best constituted safeguards to be secured by such legislation.

Perhaps, of all the professions giving much to the common welfare of the whole people, there is not one that has so completely forgotten the need of educating the public to the broad work done by our members in the interest of public health and safety. For hundreds of years, abroad and at home, veterinarians have given their protective influences to work that concerned public health in a great degree, and in so doing laid no responsibility upon the public in return, forgetful of the first duty, in a measure, to our fellow-man, that all safeguards, privileges, and pleasures lay on the individual added responsibilities, and these must be discharged by better recognition and concessions to the source-giving power from which they proceed. Our members have gone on, and are still pursuing these same lines, increasing their zeal and interest in broader sanitary safeguards, in conserving the vested moneyed interests of a large proportion of our people, taxing their whole minds and physical powers in the field, the laboratory, the public highway, the farm, the breeding establishment, the public shops, and common households, to better protect the whole people in the field of preventive medicine, utterly forgetful of the higher duty of teaching the individual his responsibility, and having him at our side at all times in aiding such measures of public import that contribute to the easier attainment of these ends, and thus save to the community the value for years of the more mature mind of the brain-worker, whose years of labor, at best, are short when spent in such a field of uncertainty as medicine has proved for centuries. I wish it were possible for me, through your important organization, to speak to every veterinarian in our land of the great importance of educating public opinion, and how hard must be the labor of a few of our profession in every community in gaining legislation that should be conceded to us by a common impulse of the people, were they properly educated to a true conception of our work. How best this organization can remedy this widespread defect of our education, I leave to the more mature consideration of many of our members, whose painful experiences and unceasing efforts are too well known to many of us to need further comment from me.

Want of a central directing power is much felt in all such efforts, and I know of no one line of work where this organization could be so great a force as in accepting in full the responsibility of directing the whole work of legislation. Neither nation nor State in any fitting measure has given to your efforts in years gone by worthy recognition of the great interests you have protected. Name me the school in the whole of North America that has ever fittingly compensated a single promoter or teacher? Name me a State, county, city, or town that has not received, through your efforts in educating men for the profession, a greater return in better protected invested monetary interests (not to speak of their work in protecting public health) than you have received in return. Your schools are without proper financial support; without national or State recognition; the public, ignorant of their importance, leave their wealth to pass into other channels, often to be worse than wasted, while your labors and difficulties become more perplexing and unremunerative. I am optimistic enough to believe that this need not be so for any great period longer, could this organization become a great central educational bureau, with its corps of assistants all over the country. A university-extension system of work could be directed, and the public press be made to serve as a common carrier of such useful information as would best further our ends, and not in these years of advanced education have copied in some paper in every State of the Union the apparently curious information that there existed in one large city of our land a hospital for the treatment of the domesticated animals. The question of food-inspection alone is one that the average reader is more ignorant of than any other problem half as important to his own personal welfare; yet in the great work to be done, how little has been accomplished! Here and there in our great commonwealths may be found a few towns where meat- and milk-inspectors are regularly maintained, and in every instance this has resulted from individual efforts through the lay press, which could have been extended by direction through a central bureau to hundreds of points where there are to-day but single ones.

I hear some one say, If we went to the legislative halls, and asked for legislation to protect legitimate practitioners and prevent future entries into this profession save those who had received a proper college education, it would be said we had an axe to grind, a selfish motive in view. Yes, and any director or promoter of a college who could not answer this question with such force and clearness as to shame the one who presented it, and win for his cause a large measure of support, is not worthy of the place and power vested in him in such a position. Think, for a moment, what the Bureau of Animal Industry alone has done for vested money interests of our land, and show me as ill-paid a body of educated, able men the world over. These men have come from your colleges, and are you not worthy of some consideration, some support, some recognition, or some evidence of appreciation of your work?

Now, such a bureau could well direct on what lines such legislation should be formed; could well suggest the best means of securing the assistance of the daily press, and furnish to them suggested features of the public aspect of needed legislation as to interest the public reader, and thus make easier the attainment of these measures, and keep more uniform the character of such legislation, and thus serve the true interests of higher education and add to the measure of pleasure, happiness, and contentment of the greatest nation of people in the world's history.

